# Retraction: The combination of organic and inorganic fertilizers influence the weed growth, productivity and soil fertility of monsoon rice

**DOI:** 10.1371/journal.pone.0323143

**Published:** 2025-05-07

**Authors:** The PLOS One Editors

The *PLOS One* Editors retract this article [1] due to concerns about potential manipulation of the publication process. These concerns call into question the validity and provenance of the reported results. We regret that the issues were not identified prior to the article’s publication.

DG, KB, AD, SS, DM, and AH did not agree with the retraction. MS, DR, MB, VH, PV, and MHM either did not respond directly or could not be reached.
